# Global Incidence and Prevalence of Gaucher Disease: A Targeted Literature Review

**DOI:** 10.3390/jcm12010085

**Published:** 2022-12-22

**Authors:** Genaro Castillon, Shun-Chiao Chang, Yola Moride

**Affiliations:** 1 YolaRX Consultants Inc., Montreal, QC H3H 1V4, Canada; 2 Takeda Development Center Americas, Inc., Cambridge, MA 02142, USA; 3 Center for Pharmacoepidemiology and Treatment Science, Rutgers, The State University of New Jersey, New Brunswick, NJ 08901, USA

**Keywords:** Gaucher disease, incidence, prevalence, real-world data

## Abstract

Incidence and prevalence estimates for Gaucher disease (GD) are scarce for this rare disease and can be variable within the same region. This review provides a qualitative synthesis of global GD incidence and prevalence estimates, GD1–3 type-specific and overall, published in the last 10 years. A targeted literature search was conducted across multiple databases from January 2011 to September 2020, including web-based sources and congress proceedings to May 2021. Searches yielded 490 publications, with 31 analyzed: 20 cohort studies (15 prospective, 5 retrospective), 6 cross-sectional studies, 5 online reports (most from Europe (*n* = 11) or North America (*n* = 11); one multiregional). Across all GD types, incidence estimates ranged 0.45–25.0/100,000 live births (16 studies), lowest for Asia-Pacific. Incidence of GD1: 0.45–22.9/100,000 live births (Europe and North America) and GD3: 1.36/100,000 live births (Asia-Pacific only). GD type-specific prevalence estimates per 100,000 population were GD1: 0.26–0.63; GD2 and GD3: 0.02–0.08 (Europe only); estimates for GD type unspecified or overall ranged 0.11–139.0/100,000 inhabitants (17 studies), highest for North America. Generalizability was assessed as “adequate”or “intermediate” for all regions with data. GD incidence and prevalence estimates for the last 10 years varied considerably between regions and were poorly documented outside Europe and North America. Data for GD2 and GD3 were limited.

## 1. Introduction

Gaucher disease (GD) is among the most prevalent of the lysosomal storage disorders (LSDs), a group of over 70 inherited metabolic diseases with a combined frequency of ~1:5000 live births [1]. Specifically, the incidence of GD in the general population has been estimated previously at between 0.39 and 5.80 per 100,000 live births [2], and also at 1.5 (95% confidence interval [CI] 1.0–2.0) per 100,000 live births [3]. Prevalence estimates for GD per 100,000 population included the range from 0.70 to 1.75 [2] and 0.9 (95% CI 0.7–1.1) [3].

GD is an autosomal recessive LSD caused by mutations in the *GBA1* gene encoding the glucosylceramide-degrading enzyme β-glucocerebrosidase [4]. Accumulation of glucosylceramide in macrophages leads to a range of clinical manifestations of varying severity and age of onset, classified into three clinical types: GD1–3 [5]. Across the broad phenotypic spectrum of GD, clinical presentations can include splenomegaly, hepatomegaly, and blood and bone abnormalities; these are typical of GD1 (the type affecting > 90% of patients with GD from Europe and North America). Neurologic symptoms are distinctive of GD2, an acute and severe neurologic form of the disease, and GD3, a chronic neurologic form [6,7].

Delayed diagnosis or misdiagnosis of GD commonly occurs on account of the complex clinical presentation of this multisystem disorder, coupled with a lack of awareness about this rare disease [8,9,10]. Patient outcomes can be improved by timely administration of enzyme replacement or substrate reduction therapies early in the disease course [11,12,13]; conversely, delays to the initiation of appropriate therapy can lead to irreversible health damage [10,13].

Prevalence reflects the estimated number of patients with GD in the population of a country/region at a given time (point prevalence) or period (period prevalence), and incidence is the occurrence of new cases of GD occurring in a population over a particular period of time.

GD incidence is associated with ethnicity and is known to be higher in particular populations, such as those of Ashkenazi Jewish descent (estimated at 1 in 450 births for GD type 1) and a population from the Norrbotten and Västerbotten geographical areas of Sweden (estimated at 1 in 50,000 births for GD type 3) [9,14,15]; however, GD affects all ethnic groups and prevalence is likely to be underestimated in many countries [16,17]. Newborn screening programs were developed for several LSDs, aiming to achieve earlier disease detection with a view to improving long-term patient outcomes [18,19,20]. Heterogeneity among the epidemiological estimates for GD can be a result of studies focusing on local ethnic groups or on particular health-seeking study populations.

There is a need for a better understanding of the global incidence and prevalence of GD, together with an evaluation of incidence rates for specific ethnic populations found within each geographic region. This will help achieve better forecasting of disease burden and improve the evaluation of treatment provision. The objective of this targeted review was to provide a qualitative synthesis of global GD incidence and prevalence estimates by region, overall, and by disease type, published in the last 10 years.

## 2. Methods

### 2.1. Literature Searches

The methodology for this targeted literature search was derived from the National Academy of Medicine standard [21]. Publications in English indexed in the MEDLINE and EMBASE databases were searched from 1 January 2011 to 30 September 2020. The search strategies combined search terms for the population of interest (patients with GD of any type) with outcomes of interest (incidence and prevalence). The geographical scope of the review was worldwide, although there was a particular focus on the GD3 type in the Asia-Pacific region when outputs were screened; however, this focus did not influence the search strategy.

If no recent, generalizable estimates were found for the parameter (incidence or prevalence) and region, pragmatic searches were conducted to identify additional sources where needed. If no estimates were found or if the generalizability of available estimates was graded as “poor”, pragmatic searches were conducted for data from which incidence and/or prevalence estimates could be derived (i.e., studies reporting on the number of patients with GD and the time period). Additional sources included: OpenGrey (the system for information on the gray literature in Europe), The Grey Literature Report (produced by the New York Academy of Medicine), and Orphanet (portal for rare diseases and orphan drugs in Europe).

The search strategy used was based on disease of interest (“Gaucher disease” OR “Gaucher disease type 1” OR “Gaucher disease type 2” OR “Gaucher disease type 3”) combined with outcomes of interest (“incidence” OR “prevalence”). Other web-based sources were also searched, which included relevant societies and congress proceedings (last date searched: 6 May 2021) as well as the citations from retrieved publications (search methodology termed “snowballing”). Country-specific incidence or prevalence was estimated using the number of patients with GD and size of catchment population matched for the time period. Where possible, estimates were standardized to per 100,000 for comparison purposes.

### 2.2. Study Selection

Publications retrieved from searches were screened for eligibility by a single assessor in a two-stage process based on prespecified eligibility criteria (Table 1). Stage 1 screening: after removal of duplicates, title and abstracts from the literature search outputs (published from 2011 onwards) were manually screened against the study eligibility criteria (Table 1A). Stage 2 screening: search outputs retained after Stage 1 screening underwent in-depth full-text review to confirm eligibility using Patient, Intervention, Comparator, Outcomes, Time period, Setting (PICOTS)-based criteria (Table 1B).

The generalizability of incidence and prevalence estimates to a region was graded (“adequate,” “intermediate,” or “poor”) on the basis of prespecified criteria related to population coverage, the number and size of countries within a given region, and the characteristics and size of the study population (Table 2).

Following the screening process, eligible publications underwent standardized data extraction by a single assessor using a data extraction form (the initial pilot was carried out by two independent assessors). Quality control checks for screening and data extraction were performed by a second assessor on a random sample of 10% of studies. A qualitative and narrative synthesis of estimates was provided for each epidemiologic parameter of interest (incidence and prevalence). Findings were reported according to GD type and country or region of interest, when available. There was no pooling of estimates or derivation of weighted averages.

## 3. Results

### 3.1. Search Outputs

**Figure 1:**
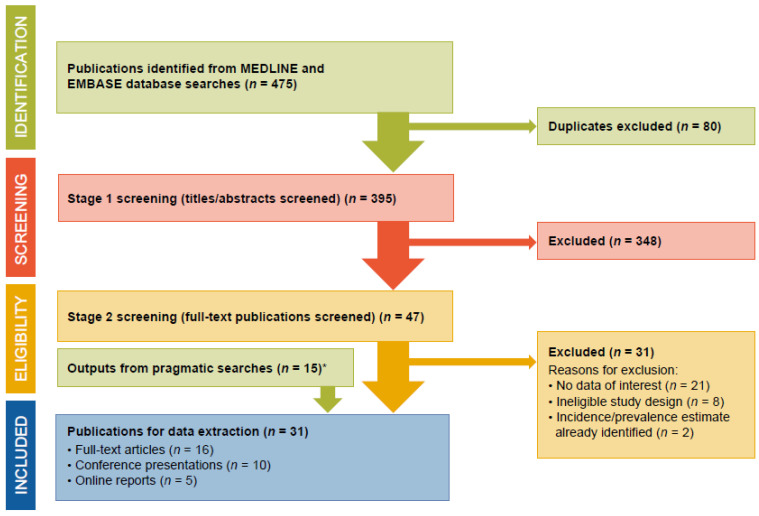
PRISMA flow chart. * Pragmatic searches yielded 13 web-based sources and two additional outputs identified from snowballing search methodology (referring to the use of reference lists or citations from identified sources to find additional sources). PRISMA, Preferred Reporting Items for Systematic Reviews and Meta-Analyses.

Stepwise screening of outputs from the literature search, with reasons for exclusion, were documented in a Preferred Reporting Items for Systematic Reviews and Meta-Analyses (PRISMA) flow chart (Figure 1).

Initial searches identified 475 publications from MEDLINE and EMBASE; 395 underwent Stage 1 eligibility screening following duplicate removal, and 47 were retained for in-depth Stage 2 eligibility screening, which excluded a further 31 publications. With 15 additional sources (13 web-based, two identified by snowballing search methodology) identified from pragmatic searches, a total of 31 outputs were retained for data extraction: 20 cohort studies (15 prospective and five retrospective), six cross-sectional studies, and five online reports (Figure 1). Following quality control checks of the publication screening and data extraction processes, inter-assessor agreement was 92.5%.

Publications most commonly involved ad hoc data collection in prospective cohort studies (*n* = 12, 38.7%) and disease registries (*n* = 12, 38.7%). The majority of studies were from Europe (*n* = 11, 35.5%) or North America (*n* = 11, 35.5%), and one study was multiregional [22].

### 3.2. GD Incidence

In the studies that were identified and included in this review, the incidence was defined as either the number of new diagnoses of the disease during the study period divided by the total births in the same period (i.e., birth incidence), or the number of newly diagnosed cases among hospital visits or in the general population. Global incidence estimates for any GD type per 100,000 live births ranged from 0.45 to 25.0 (data from 13 prospective studies [23,24,25,26,27,28,29,30,31,32,33,34,35], two retrospective cohort studies [36,37], and one newsletter from a National GD registry [38]). Most sources covered Europe (*n* = 7, 41.2%) and North America (defined as the USA and Canada; *n* = 7, 41.2%), then the Asia-Pacific region (*n* = 2, 11.8%). For the majority of studies, incidence estimates appeared to be derived from a general population of mixed ancestry rather than from specific populations, except for two estimates from the USA, one confirmed as being from a population of Ashkenazi Jewish ethnicity (22.9) [33] and another from a health-seeking population (20.0) [30]. The majority of incidence estimates were for unspecified GD, five estimates were GD type-specific, and one was for GD overall. Data from newborn screening programs contributed 13 of the 17 estimates of GD incidence from 16 studies: four out of seven estimates from Europe (4.5 [23], 5.76 [31], 7.5 [34], and 7.82 [32]), seven out of eight estimates from North America (0.45 [25], 1.42 [27], 1.59 [35], 2.27 [25], 2.29 [28], 22.9 [33], and 25.0 [24]) and both incidence estimates from Asia-Pacific (1.24 [29] and 1.36 [26]). In general, lower GD incidence estimates were reported in the Asia-Pacific region compared with Europe and North America. Incidence estimates from North America and Europe were considered of intermediate generalizability to those regions, while estimates from Asia-Pacific were of adequate generalizability.

**Figure 2:**
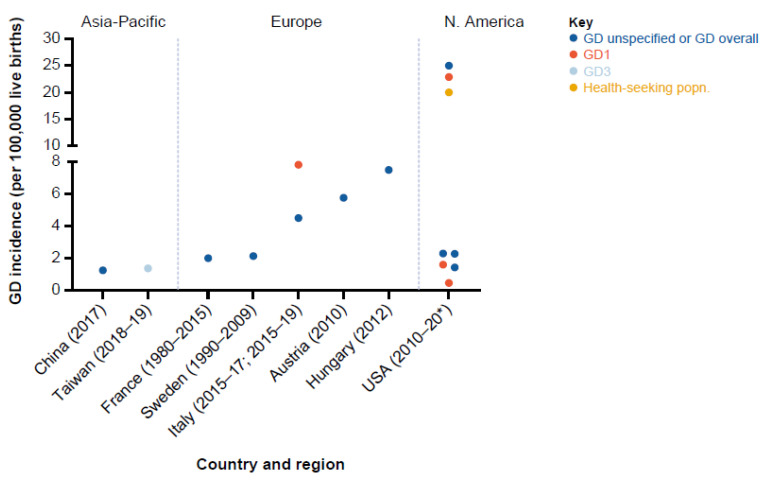
Summary of GD incidence estimates by country and region. GD unspecified refers to absence of any mention of whether study targeted GD overall or a given type. * For the eight data points from the USA (in ascending order), the corresponding time periods of study were: 2014–2016; 2017 ^†^; 2016 ^†^; 2014–2016; 2013; 2020 ^†^; 2013–2017; 2010–2011. ^†^ Year of publication of the study.

Incidence estimates by GD type were all based on data from newborn screening programs and included estimates for GD1 incidence (0.45–22.9/100,000 live births) from four studies: three from North America and one from Europe. The estimate for GD3 (1.36/100,000 live births) was from one study in the Asia-Pacific region [26] (Table 3, Table 4 and Table 5, Figure 2).

#### 3.2.1. Europe

Incidence estimates for Europe (from seven studies) ranged from 2.0 to 7.82/100,000 live births for GD (unspecified GD type or overall). Three out of seven incidence estimates (5.76 [31], 7.50 [34], and 7.82 [32]) contributing to the range were from newborn screening programs and the generalizability of the estimates was graded as intermediate because data were included from three out of the five designated countries: France, Italy, and Spain (Table 3). GD1 incidence (4.5/100,000 live births [23]) was reported by one study from Northern Italy from a newborn screening program.

#### 3.2.2. North America

Incidence estimates for any GD type (eight estimates from seven studies) were highly variable, ranging from 0.45 to 25.0/100,000 live births; seven out of eight incidence estimates were from newborn screening programs. GD1 incidence (0.45 to 22.9/100,000 live births) was reported by three studies from newborn screening programs. Incidence estimates for GD (excluding GD type-specific estimates) were 1.42 to 25.0/100,000 live births; four out of six estimates (1.42 [27], 2.27 [25], 2.29 [28], and 25.0 [24]) contributing to this range were from newborn screening programs. The generalizability of the estimates to North America was graded as intermediate as the data included were from the USA and not Canada (Table 4).

#### 3.2.3. Asia-Pacific

The incidence estimate for GD (unspecified) from one study in China was 1.24/100,000 live births [29]; GD3 incidence was reported by one study from Taiwan (1.36/100,000) [26]. Both estimates were from newborn screening programs and generalizability of the estimates was graded as adequate (data from China included) [26,29] (Table 5).

### 3.3. GD Prevalence

**Figure 3:**
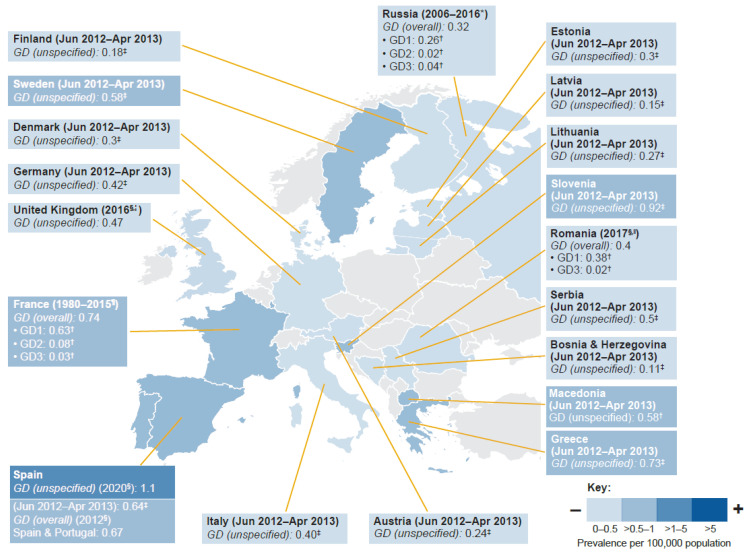
Prevalence estimates for GD: Europe. Data sourced from cross-sectional studies unless otherwise stated. One study contributing prevalence data was multiregional. Some studies reported prevalence estimates for more than one GD type. GD unspecified refers to absence of any mention of whether study targeted GD overall or a given type. Estimates from source reference [22] except France [37]; Spain GD unspecified (2020) [38]; Spain and Portugal GD overall (2012) [46]; Romania [44]; Russia [47]; UK [21]. * Retrospective cohort study of the Russian population aged > 18 years 2006–2016 [47]. ^†^ Estimates were calculated using the reported prevalence and distribution of GD types. ^‡^ Estimates were calculated using the country population size during the study period. ^§^ Year of publication. ^∥^ Cross-sectional study of the Romanian population in 2017 [44]. ^¶^ Retrospective cohort study of the French population in 1980–2015 [37]. ^¦^ Society report of UK population in 2016 [21].

**Figure 4:**
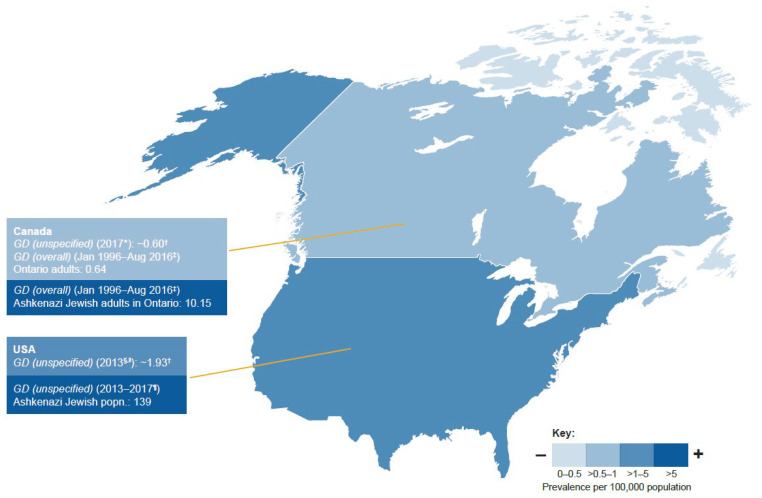
Prevalence estimates for GD: North America. GD unspecified refers to absence of any mention of whether study targeted GD overall or a given type. * Source: Cerdelga^®^ notice of refusal in Quebec population in 2017 [51]. ^†^ Estimates were calculated using the country population size during the study period. ^‡^ Retrospective cohort study in adult Ontario population in 2016 [43]. ^§^ Estimates were calculated using the reported prevalence and distribution of GD types. ^∥^ Source: Physician’s guide to Gaucher Disease from NORD US population in 2013 [50]. ^¶^ Prospective cohort of Ashkenazi Jewish students participating in an at-home national Jewish genetic disease screening initiative [40].

**Figure 5:**
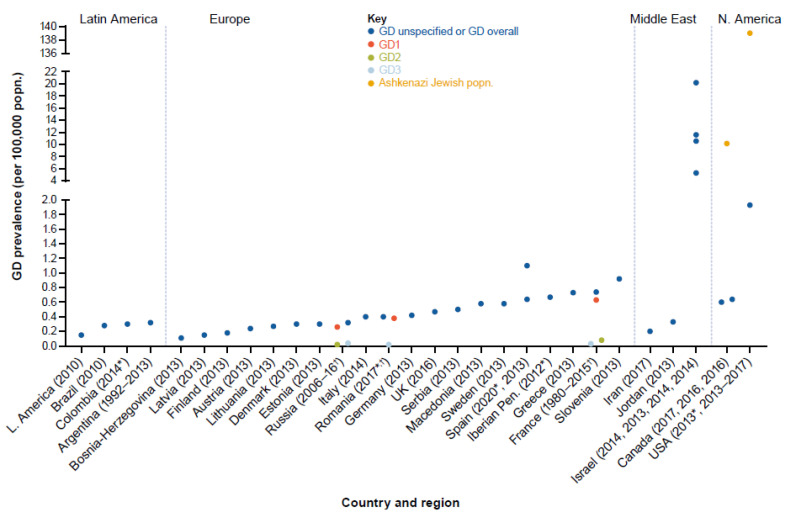
Summary GD prevalence estimates by country and region. GD unspecified refers to absence of any mention of whether study targeted GD overall or a given type. * Year of publication. ^†^ Study reported prevalence estimates for more than one GD type and GD overall.

All of the studies that were identified and included in this review examined standard prevalence as the number of patients with GD per 100,000 general population. Global prevalence estimates for any GD type per 100,000 population ranged from 0.02 to 139.0 (data from two prospective studies [39,40], four retrospective cohort studies [37,41,42,43], six cross-sectional studies [22,42,44,45,46,47], and five reports [38,48,49,50,51]). Most sources (*n* = 6) provided prevalence estimates for European populations, followed by those from North America (*n* = 4), Latin America (*n* = 3), and the Middle East (*n* = 3); one study provided multiregional data [22]. There were no prevalence estimates for the Asia-Pacific region. The majority of prevalence estimates were for unspecified GD; there were six estimates for GD overall and eight GD type-specific estimates. The highest single prevalence estimate (139.0) was for an Ashkenazi Jewish population in North America [40], whereas the lowest was 0.02 from one study on GD2 [47] and one study for GD3 [44], both from Europe. GD type-specific prevalence estimates were only available for Europe (Figure 3, Figure 4 and Figure 5 and Supplemental Tables S1 and S2).

#### 3.3.1. Europe

Estimates for the prevalence of any GD type per 100,000 population ranged from 0.02 to 1.1 (seven publications). Regional generalizability of the estimates was graded as adequate because they included all of the five prespecified countries (France, Germany, Italy, Spain, and UK). Prevalence estimates for unspecified GD (excluding estimates for GD1–3) ranged from 0.11 to 1.1 per 100,000 population. Prevalence of GD1 ranged from 0.26 [47] to 0.63 [37] per 100,000 population. The lowest prevalence estimates were type-specific for GD2: 0.02 [47] to 0.08 [37] and GD3: 0.02 [44,47] to 0.04 [47] (Figure 3).

#### 3.3.2. North America

Prevalence estimates for any GD (from four studies) ranged from 0.60 to 139/100,000 population. The two highest estimates (139.0 [40] and 10.15 [43]) were both from Ashkenazi Jewish populations: one from a US prospective cohort study reporting results from saliva-based GD screening of Ashkenazi Jewish adults [40] and the second from a retrospective chart review of adults with at least one GD specialist consultation from a GD referral center in Ontario, Canada with GD detection using β-glucocerebrosidase activity in leukocytes or fibroblasts [43] (Figure 4). Generalizability of the estimates was graded as adequate because data were included from Canada and the USA. Prevalence of GD that was unspecified or overall (excluding the two estimates from Ashkenazi Jewish populations) ranged from 0.60 [51] to 1.93 [50]/100,000 population.

Prevalence estimates were also available for Latin America: 0.15 [41] to 0.32 [39] (four estimates from three studies [39,41,48]) and were considered of adequate generalizability (data included from three of the four named countries: Argentina, Brazil, Colombia; no data from Mexico). Prevalence estimates for the Middle East were 0.20 [45] to 20.2 [52] (six estimates from four studies [22,42,45,52]) and were of intermediate generalizability (data included from two of the four named countries: Iran and Israel; no data from Egypt or Turkey). There were no prevalence estimates for the Asia-Pacific region.

## 4. Discussion

This targeted literature review provides a global overview of GD incidence and prevalence estimates from the past 10 years, together with an evaluation of the generalizability of these estimates to each region studied. Global incidence estimates for GD overall (any GD type from 16 studies) ranged from 0.45 to 25.0 per 100,000 live births, with the data mostly derived from cohort studies in Europe and North America (two studies from Asia-Pacific). Data on GD incidence were scarce in the literature, and GD type-specific estimates in particular were found in only five studies: four for GD1, none for GD2, and one for GD3. Based on prespecified criteria, the regional generalizability of incidence estimates was considered intermediate for North America and Europe and adequate for the Asia-Pacific region.

For any GD, incidence estimates for North America (0.45–25.0) were higher than for Europe (2.0–7.82) and Asia-Pacific (1.24–1.36). The global incidence range was also higher and more variable than reported in a previous qualitative literature review (0.39 to 5.80 per 100,000 births), which included data from 10 national cohort-based studies conducted in general populations of mixed ancestry [2]. A quantitative synthesis of published data pooled from 16 studies calculated GD birth prevalence as 1.5 cases (95% CI 1.0–2.0) per 100,000 live births, with a higher value for Europe (*n* = 8 studies; 1.7 [95% CI 1.0–2.3]) compared with North America (*n* = 4 studies; 1.3 [95% CI 0.2–2.4]) [3].

After removal of two of the highest incidence estimates from North America identified from health-seeking populations [30] or those of Ashkenazi Jewish descent [33] from our study, one of the higher estimates for incidence of 25.0 per 100,000 births could not be excluded on either of these grounds. However, without this estimate, a range of 0.45–7.82 for global GD incidence would be more in line with the previous qualitative literature review [2]. Of note, the 25.0 estimate was derived from a pilot blood spot screening program for LSD in Illinois, USA, where two cases of unspecified GD were identified from sampling 8012 newborn infants over 6 months [24]. The study authors conceded that data were inconclusive for some infants and recommended second-tier testing and long-term follow-up to address high false-positive rates reported from pilot LSD screening programs [25,27,53]. The majority of estimates of GD incidence (13 of 17) were from newborn screening programs, including the three highest GD incidence estimates from Europe (5.76, 7.50, and 7.82). Variability found in the incidence estimates within regions can be attributed to data derived from specific, health-seeking populations or from studies of particular GD carrier populations being set alongside studies from the general population and data from newborn screening programs [18,19]. All these sources of variability were applicable to the data collected for North America in this review. Identification of GD in newborn screening programs was largely reliant on assays detecting reduced β-glucocerebrosidase activity in dried blood samples collected from newborn infants, determined by tandem mass spectrometry [23,31,33,34]. Other methods included liquid chromatography tandem mass spectrometry [31] and the digital microfluidic enzyme assay [28]. The methodology for identifying GD varied and was not provided for all newborn screening programs. Studies comparing different methodologies for analyzing β-glucocerebrosidase activity in dried blood specimens together with *GBA* gene sequencing of the same patient samples have highlighted several analytical variables affecting data reliability [54,55], such that a shift to GD diagnosis based on glucosylsphingosine (lyso-Gb1) measurements and *GBA* mutation analyses has been proposed [56]. General criticisms of data from pilot newborn LSD screening programs related to the reporting of high incidence rates [27] that were not predictive of disease phenotype. These were attributed to false-positive assay results, pseudodeficiency alleles (alleles that alter gene expression to produce low enzyme activity detected by assays but in the absence of clinical disease), and late-onset milder phenotypes [53].

All GD type-specific incidence estimates were from newborn screening programs. GD1 incidence was reported for Europe and North America only (0.45–22.9) and GD3 incidence for Asia-Pacific only (1.36), consistent with observed regional differences in the distribution of GD types, where GD3 is the most frequent disease type in the Asia-Pacific region. In a report published in December 2021 of data from 27 patients with GD in Thailand (seven centers) studied between 2010 and 2018, GD3 was the most common type (44.5%), followed by GD2 (40.7%) and GD1 (14.8%) [57].

When investigating average prevalence estimates there is potential for the inaccurate generalization of regional estimates, which can be distorted by estimates from specific ethnic groups and health-seeking populations. There is, therefore, a need for accurate and generalizable regional estimates applicable to mixed populations, together with a better understanding of incidence rates applicable to specific populations found within regions. Estimates of GD prevalence per 100,000 population varied considerably between regions, and there were few GD type-specific prevalence estimates that could be retrieved from publications—all three of the studies contributing GD type-specific prevalence estimates were from Europe [37,44,47]. Prevalence estimates per 100,000 population for any GD ranged from 0.02 to 139.0 from 17 studies; estimates were higher in North America (0.60–139.0) than other regions, including the Middle East (0.20–20.2, including Israel), Europe (0.02–1.1), and Latin America (0.15–0.32). The highest prevalence estimate was from a population of Ashkenazi Jewish descent in North America (139.0). The lowest prevalence estimates (0.02–0.08) were GD type-specific for GD2 and GD3, which might be expected given the poor prognosis of patients with neurologic forms of GD [58]. GD1 was the most prevalent GD type in Europe and North America, consistent with previous reporting [2,58]. The regional generalizability of prevalence estimates was considered adequate for North America, Europe, Latin America, and the Asia-Pacific region and intermediate for the Middle East (because no estimates were found for Egypt or Turkey, two of the four countries of the region with the largest population sizes). The heterogeneity of prevalence estimates within the same region could be attributed to the variable distribution of different ethnic groups, as exemplified by the range of prevalence estimates from the Middle East and North America [18,19].

When excluding GD type-specific estimates and data from Ashkenazi Jewish populations (where identified) and Israel, prevalence estimates ranged from 0.11 to 1.93, in line with the previous qualitative literature review estimate of 0.70–1.75 per 100,000 population derived from seven mixed population studies [2]. The global GD prevalence calculated from data pooled from four studies was 0.9 (95% CI 0.7–1.1) per 100,000 inhabitants [3]. Considering mixed population studies identified from our review, the lowest prevalence estimates found were for Latin America (0.15–0.32), followed by the Middle East (0.20–0.33, excluding estimates from Israel), Europe (0.11–1.1), and North America (0.60–1.93).

### Limitations

The aim of this literature review—to provide a regional synthesis of recent GD incidence and prevalence estimates—took precedence over providing an all-encompassing summary of epidemiologic data available on GD. A targeted review was conducted for the period 2011 to 2020. Most incidence and prevalence estimates were identified from publications in the scientific literature indexed in MEDLINE and EMBASE using standard keyword terms; however, pragmatic searches of web-based resources and hand-searching of reference lists were included to widen the range of data sources included. Publication bias is considered less likely when reporting data from epidemiologic versus interventional clinical studies; however, this may still contribute to the lack of data available from English language scientific publications reporting on regions outside Europe and North America. It should be noted that a large proportion of GD prevalence estimates from Europe were derived from one publication (survey of The European Gaucher Alliance members) [22]; however, estimates from Israel and Spain in this study were in line with estimates for these countries from other studies. Estimates based on voluntary membership of national patient organizations may be less comprehensive in capturing all patients with GD than other health-system based surveys. Efforts to mitigate any study selection bias in the review included a quality control assessment of the screening and data extraction process by another assessor.

The synthesis of GD incidence and prevalence estimates by region in this review highlighted significant data gaps. GD incidence was poorly documented overall, and GD type-specific estimates for incidence and prevalence were rare. Few estimates were available for GD2 and GD3. Specifically, limited epidemiologic data were available for the Asia-Pacific region, and none from India or Africa, although there are case reports of patients with GD from these countries [59,60,61,62]; a large proportion of the global population were not represented. The availability of epidemiologic data on GD is likely to reflect accessibility to healthcare, because the diagnosis of GD requires the use of techniques that are both invasive and resource intensive [62]. New technologies, such as the high-throughput digital microfluidic platform [63], may offer ways to provide inexpensive, minimally invasive disease-specific testing for LSDs in developing countries. International disease registries and treatment access programs could also improve data availability for these regions [61,64,65].

When considering the reliability and comparability of epidemiologic estimates from different studies included in this review, it should be noted that different screening platforms for GD were used across studies, few studies included data from newer genetic profiling technologies [17,57], and the types of assay and measures of accuracy were generally poorly documented. An additional caveat to the interpretation of data from newborn screening programs is that they can identify asymptomatic GD carriers, which may lead to overestimation of future disease burden in terms of number of patients experiencing clinical symptoms that will require healthcare intervention [19,53]. Recent studies—including those from biobanks investigating screening for diagnosed and undiagnosed patients with GD—have indicated that extrapolating disease frequency rates from average numbers may exaggerate the numbers with GD, particularly in populations that are stable and where mutations are at a low level [55,66]. For example, applying 1:30,000–100,000 prevalence estimates to Finland results in 60–180 more patients with GD than have currently been identified in the population, which overburdens the health service in its attempt to identify additional, non-existent patients [66].

The criteria for assessing the regional generalizability of estimates for this study have not been validated and were based on objective criteria only, such as geographical coverage of the region and countries with the largest population size. Consideration of the varying ethnic backgrounds for populations found in different regions may have been more informative, given the genetic profile of the disease.

## 5. Conclusions

This literature review maps current regional and population-specific epidemiologic estimates for GD incidence and prevalence reported in the medical literature from the last 10 years. The generalizability of incidence and prevalence estimates to regional populations with available data was graded either as adequate or intermediate. A global overview of GD incidence and prevalence estimates identified important data gaps for specific regions such as Africa and countries with large populations, including India and China. Population estimates at specific time points can provide a useful benchmark from which to monitor future changes in GD incidence and prevalence and for tracking the emergence of new genetic variants associated with GD identified by genetic profiling. In the future, new diagnostic platforms—together with international disease registries and treatment access initiatives—may help to provide more accurate regional predictions for disease burden.

## Figures and Tables

**Table 1:** Publication eligibility criteria.

A. Stage 1 Screening: Study Inclusion and Exclusion Criteria	
Inclusion Criteria	Exclusion Criteria
Studies conducted in humans	Case reports, letters to editors, editorials, opinions
Observational studies (e.g., cross-sectional, cohort, case–control, registries, case series *)	Literature reviews (systematic and non-systematic) and meta-analyses (used as reference source only)
Studies that included patients with GD (either as the study population or as a subgroup analysis)	Clinical trials, non-clinical or experimental (preclinical) studies
Studies that reported incidence and/or prevalence estimates	Studies reporting preliminary results (if later published as full text)
Studies published as full text, conference proceedings, or abstracts	
Studies published between 1 January 2011 and 6 May 2021 (date last searched) ^†^	
Search was in English but outputs in French, Spanish, German, or Italian only were also considered when necessary	
**B. Stage 2 Screening Criteria.**	
**Criteria** **(based on PICOTS)**	**Details**
Population	Patients with GD of any type
Intervention	Receiving standard of care (including substrate replacement therapy and enzyme replacement therapy)
Outcomes	GD incidence and prevalence outcomes or data from which these could be derived
Time period	Published within the past 10 years: 1 January 2011 to 6 May 2021
Setting	Real-world/observational studies

GD, Gaucher disease; PICOTS, Patient, Intervention, Comparator, Outcomes, Time period, Setting. * Cases series were considered because these are often conducted as non-comparative cohort studies. ^†^ For studies with multiple publications, the latest relevant publication was used.

**Table 2:** Rules to assess regional generalizability of estimates.

Assessment Criteria	Description
GD type	The availability of estimates for: **GD overall** (any GD or combining estimates where GD type was specified) **GD unspecified** (absence of any information on whether study targeted GD overall or a given GD type) **GD type-specific** (GD type 1–3 specific)
Number of regions collectively covered across studies	The availability of estimates for each region (North America, Europe, Asia-Pacific, Latin America, Middle East, and Africa) was determined
Countries covered	Listed countries were based on an assessment of the number and population size of countries per region: **Asia-Pacific:** If either China or India were covered, then the generalizability was considered as adequate **Europe:** If available studies collectively covered at least 4 countries among France, Germany, Italy, Spain, UK, then the findings were considered to have adequate generalizability. If only 2 or 3 of these countries were covered then generalizability was considered intermediate, and 0–1 was considered poor generalizability **North America** (includes the USA and Canada) *: If estimates were only available for either the USA or Canada, generalizability was deemed intermediate, otherwise, if both were covered both, then it was considered adequate **Latin America:** If available studies collectively covered at least 3 countries among Argentina, Brazil, Colombia, and Mexico, then the findings were considered to have adequate generalizability. If only 2 of the above-listed countries were covered, generalizability was considered intermediate, and 0–1 was considered poor **Middle East:** Generalizability was considered adequate if at least 3 countries among Egypt, Iran, Jordan, or Turkey were included, intermediate if 2 out of the 4 listed countries were included, and poor for 0 or 1 out of 4 countries **Africa:** Generalizability was considered adequate if at least 3 of the following countries were covered: Algeria or Morocco; South Africa; or any country from sub-Saharan Africa. If only some of those countries were covered then the generalizability was considered intermediate (2 out of 3 countries) or poor (0 or 1 out of 3)
Size of study population	Within a country or region, the size of the studies (collective or individual) was also considered. For guidance purposes, studies with a sample size >200 patients with GD were considered arbitrarily to be large

* Mexico was considered part of Latin America.

**Table 3:** GD incidence: Europe.

	Study Design	Study Period	Study Duration, Months	Study Population Size	Reference Population	Incidence Rate (Confirmed Cases/Screened Pts)
**France**						
Stirnemann et al. 2016 [37]	Retrospective cohort	1980–2015	-	616	Live births in corresponding years	**GD (overall)** 2.0/100,000 live births
**Sweden**						
Hult et al. 2014 [36]	Retrospective cohort	1990–2009	360	44/2,080,791	Live births in corresponding years	**GD (unspecified)** 2.13/100,000 live births
**Italy**						
Burlina et al. 2018 [23]	Prospective cohort	Sep 2015–Jan 2017	17	2/44,411	Population-based newborn screening program in North-East Italy	**GD1:** 4.50/100,000 live births
Polo et al. 2020 [32]	Prospective cohort	Sep 2015–Aug 2019	-	2/127,869	Population-based newborn screening program	**GD (unspecified):** 7.82/100,000 live births
**Austria**						
Mechtler et al. 2012 [31]	Prospective cohort	Jan 2010–Jul 2010	7	2/34,736	Population-based newborn screening program	**GD (unspecified):** 5.76/100,000 live births
**Hungary**						
Wittmann et al. 2012 [34]	Prospective cohort	2012 *	-	3/40,024	Population-based newborn screening program	**GD (unspecified):** 7.5/100,000 live births
**Spain**						
SEHH 2020 [38]	Newsletter from the Spanish registry of GD	2020 *	-	NA	Population of Spain in 2019	**GD (unspecified):** 8–10 new cases/year

GD unspecified refers to absence of any mention of whether study targeted GD overall or a given type. Criteria for grading generalizability of estimates from Europe: Adequate = four or more named countries: France, Germany, Italy, Spain, and UK; Intermediate = two or three named countries. * Year of publication. NA, not available.

**Table 4:** GD incidence: North America.

USA	Study Design	Study Period	Study Duration, Months	Study Population Size	Reference Population	Incidence Rate (Confirmed Cases/Screened Pts)
Hopkins et al. 2017 [27]	Prospective cohort	2017 *	-	4/282,500	Missouri pilot newborn screening program	**GD (unspecified)** 1.42/100,000 live births
Burton et al. 2016 [35]	Prospective cohort	2016 *	-	1/63,007	Illinois newborn screening program	**GD1:** 1.59/100,000 live births
Hopkins et al. 2015 [28]	Prospective cohort	Jan 2013–Jun 2013	6	1/43,701	Missouri newborn screening program	**GD (unspecified)** 2.29/100,000 live births
Burton et al. 2017 [25]	Prospective cohort	Nov 2014–Aug 2016	-	5/219,793	Illinois Department of Public Health in Chicago newborn screening program	**GD (unspecified):** 2.27/100,000 live births **GD1**: 0.45/100,000 live births (1/219,793)
Wasserstein et al. 2019 [33]	Prospective cohort	May 2013–Apr 2017	48	15/65,605	New York pilot newborn screening program	**GD1:** 22.9/100,000 live births ^†^
Burton et al. 2012 [24]	Prospective cohort	Nov 2010–Apr 2011	6	2/8012	Illinois pilot newborn screening program	**GD (unspecified):** 25.0/100,000 live births
Limgala et al. 2020 [30]	Prospective cohort	2020 *	-	1/5000	Patients (all ages) seeking healthcare for various health concerns: 85% African American 10% Hispanic 5% Caucasian/other	**GD (unspecified):** 20.0/100,000 healthcare-seeking patients

GD unspecified refers to absence of any mention of whether study targeted GD overall or a given type. Criteria for grading generalizability of estimates from North America: Adequate = Canada and the USA; Intermediate = Canada or the USA. * Year of publication. ^†^ All confirmed GD1 cases were of Ashkenazi Jewish descent.

**Table 5:** GD incidence: Asia-Pacific region.

	Study Design	Study Period	Study Duration, Months	Study Population Size	Reference Population	Incidence Rate (Confirmed Cases/Screened Pts)
**China**						
Kang et al. 2017 [29]	Prospective cohort	2017 *	12	1/80,855	Newborns participating in the Neonatal Screening Center of Shanghai	**GD (unspecified)** 1.24/100,000 live births
**Taiwan**						
Chien et al. 2020 [26]	Prospective cohort	Mar 2018–Apr 2019	12	1/73,743	35% of newborns in Taiwan	**GD3:** 1.36/100,000 live births

GD unspecified refers to absence of any mention of whether study targeted GD overall or a given type * Year of publication.

## Data Availability

The datasets supporting this analysis are available from the corresponding author on reasonable request.

## Supplementary Materials

The following supporting information can be downloaded at: https://www.mdpi.com/article/10.3390/jcm12010085/s1. Supplemental Table S1. GD prevalence: Europe. Supplemental Table S2. GD prevalence: North America. Table S3. PRISMA checklist.
