# Supplementary material for: Global Incidence and Prevalence of Gaucher Disease: A Targeted Literature Review
Source: J Clin Med. 2022 Dec 22;12(1):85. doi: 10.3390/jcm12010085 (PMC9821068; doi:10.3390/jcm12010085)
Supplement: Supplementary file 1 [file jcm-12-00085-s001.zip › jcm-2106844-supplementary.pdf]

Supplementary material

Supplemental Table S1. GD prevalence: Europe.

	Study Design	Study Period	Reference Population	Prevalence
		Finland		
Žnidar et al. 2014 [20]	Cross-sectional	Jun 2012–Apr 2013	Finnish population in 2013	GD (unspecified) 0.18/100,000 inhabitants*
		Austria		
Žnidar et al. 2014 [20]	Cross-sectional	Jun 2012–Apr 2013	Austrian population in 2013	GD (unspecified) 0.24/100,000 inhabitants*
		Lithuania		
Žnidar et al. 2014 [20]	Cross-sectional	Jun 2012–Apr 2013	Lithuanian population in 2013	GD (unspecified) 0.27/100,000 inhabitants*
		Denmark		
Žnidar et al. 2014 [20]	Cross-sectional	Jun 2012–Apr 2013	Danish population in 2013	GD (unspecified) 0.3/100,000 inhabitants*
		Estonia		
Žnidar et al. 2014 [20]	Cross-sectional	Jun 2012–Apr 2013	Estonian population in 2013	GD (unspecified) 0.3/100,000 inhabitants*
		Russia		
Movsisyan et al. 2017 [45]	Retrospective cohort	2006–2016	Population <18 years of age in Russia in corresponding years	GD overall: 0.32/100,000 children<br>GD1: 0.26/100,000 children‡<br>GD2: 0.02/100,000 children‡<br>GD3: 0.04/100,000 children‡

Italy				
Žnidar et al. 2014 [20]	Cross-sectional	Jun 2012–Apr 2013	Italian population in 2013	GD (unspecified): 0.4/100,000 inhabitants*
Romania				
Bucerzan et al. 2017 [42]	Cross-sectional	2017†	Romanian population in corresponding years	GD (overall): 0.4/100,000 inhabitants GD1: 0.38/100,000 inhabitants‡ GD3: 0.02/100,000 inhabitants‡
Germany				
Žnidar et al. 2014 [20]	Cross-sectional	Jun 2012–Apr 2013	German population in 2013	GD (unspecified): 0.42/100,000 inhabitants*
UK				
Gauchers Association [47]	Society report	2016†	UK population in 2016	GD (unspecified): 0.47/100,000 inhabitants
Serbia				
Žnidar et al. 2014 [20]	Cross-sectional	Jun 2012–Apr 2013	Serbian population in 2013	GD (unspecified): 0.5/100,000 inhabitants*
Macedonia				
Žnidar et al. 2014 [20]	Cross-sectional	Jun 2012–Apr 2013	Macedonian population in 2013	GD (unspecified): 0.58/100,000 inhabitants*
Sweden				
Žnidar et al. 2014 [20]	Cross-sectional	Jun 2012–Apr 2013	Swedish population in 2013	GD (unspecified): 0.58/100,000 inhabitants*
Iberian Peninsular				
Giraldo et al. 2012 [44]	Cross-sectional	2012†	Total number of inhabitants	GD (overall): 0.67/100,000 inhabitants

			in the Iberian Peninsula in corresponding years	
		Greece		
Žnidar et al. 2014 [20]	Cross-sectional	Jun 2012–Apr 2013	Greek population in 2013	GD (unspecified): 0.73/100,000 inhabitants*
		France		
Stirnemann et al. 2016 [35]	Retrospective cohort	1980–2015	French population in corresponding years	GD (overall): 0.74/100,000 inhabitants<br>GD1: 0.63/100,000 inhabitants‡<br>GD2: 0.08/100,000 inhabitants‡<br>GD3: 0.03/100,000 inhabitants‡
		Slovenia		
Žnidar et al. 2014 [20]	Cross-sectional	Jun 2012–Apr 2013	Slovenian population in 2013	GD (unspecified): 0.92/100,000 inhabitants*
		Spain		
Žnidar et al. 2014 [20]	Cross-sectional	Jun 2012–Apr 2013	Spanish population in 2013	GD (unspecified): 0.64/100,000 inhabitants*
SEHH 2020 [36]	Newsletter from the Spanish GD registry	2020†	Spanish population	GD (unspecified): 1.1/100,000 inhabitants

GD unspecified refers to absence of any mention of whether study targeted GD overall or a given type
*Estimates were calculated using the size of the country population.
†Year of publication.
‡Estimates were calculated using the reported prevalence and distribution of GD types.

Supplemental Table S2. GD prevalence: North America.

	Study Design	Study Period	Reference Population	Prevalence
		Canada		
INESSS 2018 [49]	(INESS, public drug payer of Quebec Province) Cerdelga® notice of refusal	2017	Quebec population in 2017	GD (unspecified): ~0.60/100,000 inhabitants*
Yu et al. 2018 [41]	Retrospective cohort	Jan 1996–Aug 2016	Adult Ontario population in 2016	GD (overall): Adults in Ontario: 0.64/100,000 Ashkenazi Jewish adults in Ontario: 10.15/100,000
		USA		
NORD 2013 [48]	Physician's Guide to Gaucher Disease from NORD	2013†	US population in 2013	GD (unspecified): ~1.93/100,000 inhabitants*
Grinzaid 2017 [38]	Prospective cohort	2013–2017	Ashkenazi Jewish students participating in an at-home national Jewish genetic disease screening initiative	GD (unspecified): 139/100,000 Ashkenazi Jewish population

GD unspecified refers to absence of any mention of whether study targeted GD overall or a given type
*Estimates were calculated using the size of the country population.
†Year of publication.

Table S3. PRISMA checklist

Section/topic	#	Checklist item	Reported on page #
TITLE			
Title	1	Identify the report as a systematic review, meta-analysis, or both.	1
ABSTRACT			
Structured summary	2	Provide a structured summary including, as applicable: background; objectives; data sources; study eligibility criteria, participants, and interventions; study appraisal and synthesis methods; results; limitations; conclusions and implications of key findings; systematic review registration number.	1, no reg. # for SLR
INTRODUCTION			
Rationale	3	Describe the rationale for the review in the context of what is already known.	1-2
Objectives	4	Provide an explicit statement of questions being addressed with reference to participants, interventions, comparisons, outcomes, and study design (PICOS).	2-3
METHODS			
Protocol and registration	5	Indicate if a review protocol exists, if and where it can be accessed (e.g., Web address), and, if available, provide registration information including registration number.	Protocol available on request
Eligibility criteria	6	Specify study characteristics (e.g., PICOS, length of follow-up) and report characteristics (e.g., years considered, language, publication status) used as criteria for eligibility, giving rationale.	2-3 Tables 1 and 2
Information sources	7	Describe all information sources (e.g., databases with dates of coverage, contact with study authors to identify additional studies) in the search and date last searched.	2-4
Search	8	Present full electronic search strategy for at least one database, including any limits used, such that it could be repeated.	2-4
Study selection	9	State the process for selecting studies (i.e., screening, eligibility, included in systematic review, and, if applicable, included in the meta-analysis).	2-3 Tables 1 and 2
Data collection process	10	Describe method of data extraction from reports (e.g., piloted forms, independently, in duplicate) and any processes for obtaining and confirming data from investigators.	2-4
Data items	11	List and define all variables for which data were sought (e.g., PICOS, funding sources) and any assumptions and simplifications made.	2-4

Risk of bias in individual studies	12	Describe methods used for assessing risk of bias of individual studies (including specification of whether this was done at the study or outcome level), and how this information is to be used in any data synthesis.	2-4
Summary measures	13	State the principal summary measures (e.g., risk ratio, difference in means).	2-4
Synthesis of results	14	Describe the methods of handling data and combining results of studies, if done, including measures of consistency (e.g., I^2) for each meta-analysis.	2-4
Risk of bias across studies	15	Specify any assessment of risk of bias that may affect the cumulative evidence (e.g., publication bias, selective reporting within studies).	NA
Additional analyses	16	Describe methods of additional analyses (e.g., sensitivity or subgroup analyses, meta-regression), if done, indicating which were pre-specified.	NA
RESULTS			
Study selection	17	Give numbers of studies screened, assessed for eligibility, and included in the review, with reasons for exclusions at each stage, ideally with a flow diagram.	Figure 1
Study characteristics	18	For each study, present characteristics for which data were extracted (e.g., study size, PICOS, follow-up period) and provide the citations.	5-12<br>Tables 3–5 and Figure 3 and 5 Supplementary Tables 1 and 2
Risk of bias within studies	19	Present data on risk of bias of each study and, if available, any outcome level assessment (see item 12).	NA
Results of individual studies	20	For all outcomes considered (benefits or harms), present, for each study: (a) simple summary data for each intervention group (b) effect estimates and confidence intervals, ideally with a forest plot.	NA
Synthesis of results	21	Present results of each meta-analysis done, including confidence intervals and measures of consistency.	NA
Risk of bias across studies	22	Present results of any assessment of risk of bias across studies (see Item 15).	NA
Additional analysis	23	Give results of additional analyses, if done (e.g., sensitivity or subgroup analyses, meta-regression [see Item 16]).	NA
DISCUSSION			
Summary of evidence	24	Summarize the main findings including the strength of evidence for each main outcome; consider their relevance to key groups (e.g., healthcare providers, users, and policy makers).	13-16

Limitations	25	Discuss limitations at study and outcome level (e.g., risk of bias), and at review-level (e.g., incomplete retrieval of identified research, reporting bias).	17-18
Conclusions	26	Provide a general interpretation of the results in the context of other evidence, and implications for future research.	19
FUNDING			
Funding	27	Describe sources of funding for the systematic review and other support (e.g., supply of data); role of funders for the systematic review.	19
